# Strategies to Mitigate Enteric Methane Emissions from Ruminant Animals

**DOI:** 10.4014/jmb.2202.02019

**Published:** 2022-03-05

**Authors:** Tenzin Tseten, Rey Anthony Sanjorjo, Moonhyuk Kwon, Seon-Won Kim

**Affiliations:** Division of Applied Life Science (BK21 Four), ABC-RLRC, PMBBRC, Gyeongsang National University, Jinju 52828, Republic of Korea

**Keywords:** Global warming, methane, ruminants, rumen microbiome, methanogenesis, direct-fed microbials

## Abstract

Human activities account for approximately two-thirds of global methane emissions, wherein the livestock sector is the single massive methane emitter. Methane is a potent greenhouse gas of over 21 times the warming effect of carbon dioxide. In the rumen, methanogens produce methane as a by-product of anaerobic fermentation. Methane released from ruminants is considered as a loss of feed energy that could otherwise be used for productivity. Economic progress and growing population will inflate meat and milk product demands, causing elevated methane emissions from this sector. In this review, diverse approaches from feed manipulation to the supplementation of organic and inorganic feed additives and direct-fed microbial in mitigating enteric methane emissions from ruminant livestock are summarized. These approaches directly or indirectly alter the rumen microbial structure thereby reducing rumen methanogenesis. Though many inorganic feed additives have remarkably reduced methane emissions from ruminants, their usage as feed additives remains unappealing because of health and safety concerns. Hence, feed additives sourced from biological materials such as direct-fed microbials have emerged as a promising technique in mitigating enteric methane emissions.

## Introduction

**Fig. 1:**
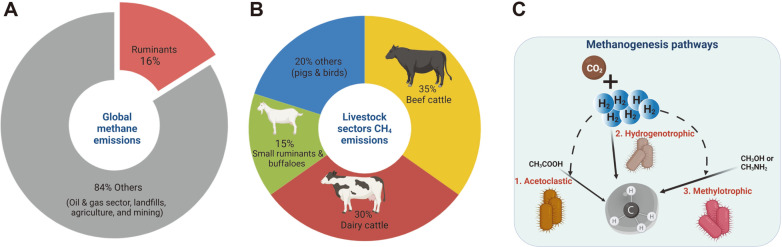
Global methane emissions and methanogenesis in rumen. **A.** Global methane emissions contributed by the ruminants and other sectors. **B.** Contributions of different animal species including beef cattle, dairy cattle, small ruminants and buffalos, and other animals such as pigs and birds to the total methane emissions from livestock. **C.** Methanogenesis pathways in the rumen.

Anthropogenic activities account for approximately two-thirds of global methane emissions [1], wherein 41%is attributable to agricultural activities which involve ruminant enteric fermentation, manure management, and rice cultivation. Around 16% of the global methane emission is contributed by ruminant animals (Fig. 1A). Within the agricultural sector, 73% of the methane emission comes from livestock [2], majorly represented by beef (35%) and dairy (30%) cattle, with only 15% from small ruminants and buffalos (Fig. 1B) [3, 4]. The United Nations (UN) has estimated that the world’s population will reach 9.8 billion in 2050 and 11.2 billion in 2100 [5], along with an increasing demand for milk and meat products by 1.04 million tons and 465 million tons, respectively [6]. As the demand for ruminant livestock increases, it results in higher methane production, accelerating global warming in the process inevitably [7].

Methane is the second most abundant greenhouse gas [8], with the potency to trap infrared radiation in the atmosphere and raise global temperature by over 21 times the ability of CO_2_ [9, 10]. It also translates to 2-12% of gross energy lost as eructed methane from animal feed [11, 12], which could have been used to boost animal productivity. Therefore, a reduction in enteric methane emission from the ruminants could improve animal performance while assuring long-term agricultural sustainability [13].

## Methanogenesis in Ruminant Animals

Ruminants are cloven-hoofed mammals of the Artiodactyla order, with domesticated cattle, sheep, and goats comprising 95% of the total ruminant population [14]. They obtain their food by browsing or grazing, subsisting on plant material using their specialized digestive system [15] with a sophisticated symbiotic web of microorganisms [16]. The digestive system of ruminants consists of four compartments - rumen, reticulum, omasum, and abomasum [17].

In the rumen, the intricate community of bacteria (10^10^-10^11^ cells/ml), ciliate protozoa (10^4^-10^6^ cells/ml), methanogenic archaea (10^6^-10^8^ cells/ml), and fungi (10^3^-10^6^ cells/ml) synthesizes enzymes which breakdown complex macromolecules derived from feed [18, 19]. This fermentative process produces short volatile fatty acids (SVFAs) and microbial crude protein, which is an essential source of energy and protein for the host, while the rumen provides the microbes a suitable environment for survival and growth [16, 20]. Acetate (~65%), propionate (~20%), and butyrate (~15%) form the major part of SVFAs in the anaerobic rumen fermentation, which supplies 80% of the animal’s total energy requirement [21]. Subsequently, methanogens in the gastrointestinal tract produce methane as a by-product of anaerobic fermentation [22].

Methanogens can be classified into three clades based on the substrate it utilizes: methane derivatives (methylotrophic), H_2_/CO_2_ (hydrogenotrophic), and acetate (acetoclastic) as shown in Fig. 1C [23, 24]. Among all groups, hydrogenotrophic methanogenesis from the substrate H_2_/CO_2_ is the main route for hydrogen disposal, where CO_2_ acts as a hydrogen sink in an anaerobic environment [25]. Likewise, nitrate and sulfate can also act as hydrogen sinks as the nitrate/sulfate reduction pathway is more thermodynamically favorable [26]. However, since its concentration in the rumen is low, this limits the rate of electron flow towards the sulfate/nitrate reduction pathway diverting the majority of H_2_ towards methane formation. Hence, methanogenesis is the most effective way of eliminating hydrogen in the rumen to allow the fermentation process to continue. This rationale emphasizes the role of rumen methanogens as a crucial target in various methane mitigating emission strategies [24].

Moreover, intercellular H_2_ transfer between methanogens and the fermentative community of protozoa, fungi, and bacteria regulates the H_2_ levels in the rumen, as traces of H_2_ have been reported to inhibit hydrogenase activity, negatively affecting carbohydrate oxidation [25, 27, 28]. Overall, the rumen fermentation process is regulated by the interspecies transfer of hydrogen between microbes and its intracellular flow into competing metabolic pathways [29].

## Mitigation Strategies

**Fig. 2:**
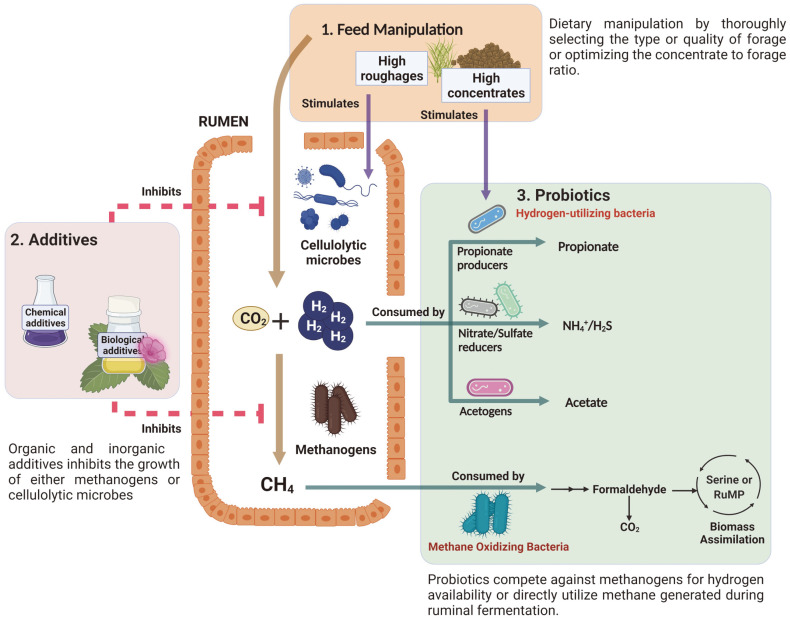
Strategies to mitigate methane emissions from ruminant animals. Feed manipulation, supplementation of additives, and probiotics. Light brown line (flow of rumen fermentation), pink line (inhibition), purple line (stimulation), and green line (consumption).

Since the 1950s, researchers have endeavored to adopt diverse strategies in minimizing enteric methane emissions. Several approaches have proved successful and shown exceptional results in reducing enteric methane emissions while improving animal productivity, but they are expensive and carry environmental and human health risks. So, it is crucial to understand existing techniques and create better solutions towards abating ruminant methane emissions (Fig. 2).

### I. Mitigation through Feed Manipulation

Dietary manipulation by changing the feed composition remains the most straightforward and inexpensive approach to lessen enteric methane levels [30, 31]. This strategy alone could curtail up to 70% of ruminant methane emissions, depending on the method or nature of the nutritional intervention [32, 33].

The predominant approach is to change the type or quality of forage or adjust the concentrate to forage ratio in the feed. The younger plants containing higher fermentable carbohydrates, less non-digestible fiber (NDF), and lower C:N ratio makes up for high-quality forage, ensuring higher digestibility and passage rate, which can direct rumen fermentation towards propionate [34, 35]. Since propionate also serves as an alternative H_2_ sink, increased propionate production leads to less H_2_ available for methanogenesis [36]. However, forage alone is not enough to enhance animal performances as concentrates are usually added to feed in different proportions as it contains fewer cell walls and readily fermentable carbohydrates (starch and sugar) [37, 38]. It has been observed that when 35% or 60% concentrate is added to feed, CH_4_ production decreases, accompanied by enhanced productivity [49]. Conversely, many groups have reported that high levels of concentrates could elevate lactic acid and volatile fatty acids (VFAs) concentration in the rumen, which contributes to health disorders such as subacute ruminal acidosis (SARA) [39, 40].

### II. Mitigation through Additives

In general, additives are added to feed consisting of either inorganic or organic compounds or direct-fed probiotics. These additives either specifically inhibit methanogens or alter the metabolic pathways leading to a reduction of the substrate for methanogenesis [30, 41].

### Ionophores

In 1975, the United States FDA approved ionophores as a cattle feed supplement [42]. Ionophores benefit animal metabolism by enhancing the efficiency of energy metabolism, improving ruminal nitrogen metabolism while reducing the risk of bloating and acidosis [43, 44]. Commercially available ionophores such as monensin (Rumensin), lasalocid (Bovatec), salinomycin (Bio-cox, Sacox), and laidlomycin (Cattlyst) are used widely across many countries including Australia, Argentina, Brazil, Canada, New Zealand, South Africa, and the United States. It is used to manipulate ruminal fermentation, improving feed efﬁciency as it has been reported to modulate the ratio of propionic to acetic acid production [45, 46], resulting in body weight gain [47]. In addition, there is also a pronounced reduction of proteolysis in the rumen, decreasing ammonia as a by-product while increasing the total flow of protein into the small intestine for absorption [48].

Ionophores also act as antimicrobials capable of disrupting the ion concentration gradient (Ca^2+^, K^+^, H^+^, Na^+^) across specific microbial membranes, causing them to enter a futile ion cycle providing a competitive advantage for specific microbes at the expense of others [49, 50]. This carboxylic polyether compound preferentially inhibits gram-positive bacteria that produce lactate, acetate, butyrate, formate, and hydrogen as end products, thereby reducing the hydrogen availability for methanogens [48]. Guan *et al*. [49] reported supplementation of ionophores correlated with a nearly 80% decrease in the ciliate protozoal population and lower methane generation in Angus yearling steers [51]. Similarly, Odongo *et al*. [47] observed over a 9% reduction in methane production, which was sustained for six months when fed with 24 mg of Rumensin Premix/kg of dry matter in lactating dairy cows.

Even though ionophores can reduce methane production, they also seem to impair dry matter intake (DMI) in both dairy cows and beef steers [52]. It also has shown that the effect of ionophores wanes over time due to the adaptation by ciliate protozoa [52, 53] and the development of resistance in succinate and propionate producing bacteria [54].

### Methanogenesis Inhibitors

The methyl-coenzyme M reductase (McR) plays a crucial role in anaerobic methanogenesis [55]. It catalyzes the final step of methane metabolism involving a methyl-transfer reaction to coenzyme M (HS-CoM or 2-mercaptoethanesulfonic acid), the electron donor coenzyme F430 containing nickel (active: Ni^+^ or inactive: Ni^+2^), reducing the substrate methyl-CoM releasing methane in the process [56, 57]. Disrupting any of these series of steps is the primary mode of several halogenated and nitro-derivatives of hydrocarbons, fatty acids, and alcohols.

For instance, halogenated, sulfonated compounds such as bromoethane sulfonate (BES) and bromopropanesulfonic acid (BPS) structurally mimic CoM (2-mercaptoethanesulfonic acid), reducing in vitro methane emissions from 70% [58] up to 80% without sacrificing organic matter digestibility and VFA concentrations [59, 60]. Chloroform also decreased the methane production by 30% (g/kg) when fed to cattle at 6-7% w/w, significantly affecting *Methanobrevibacter* and *Methanosphaera* species [61]. In contrast to other halogenated derivatives, it appears to disrupt the cobamide-dependent methyl-transferase step of the methanogenesis pathway.

A nitro derivative, 3-NOP (3-Nitrooxypropanol), also acts as a structural analog of Methyl-coenzyme M, which competitively binds to the active site of McR and its ability to oxidize the cofactor Ni^+^, thereby inactivating McR [62]. As microbes can tolerate nitro toxins from nitro compounds, daily weight gain (DWG) increased, and the DMI, milk production and digestibility remain unaffected in both Holstein cows and beef cattle [63-66]. Enteric methane emissions were diminished by 20% to 60%, depending on the method or duration of supplementation. Similar nitro compounds such as 3-nitrooxypropanol, nitroethane, and 3-nitropropionic acid are also being investigated [67].

Overall, the remarkable inhibitory capacity of nitro and halogenated derivatives gradually diminishes as resistant microbes steadily replace sensitive microbes [68]. Moreover, the significant reduction in methane leads to hydrogen accumulation inside the rumen with unknown long-term effects [69]. In addition, cost and safety concerns limit current practical application [69, 70, 71].

### Essential Oils and Other Plant Extracts

In recent years, more additives from biological sources have been investigated for their role in enhancing cattle performance and reducing greenhouse gas after the 2006 EU ban against antibiotics as growth promoters [72, 73].

Essential oils (EOs) are volatile and aromatic oily liquids extracted from plant materials such as flowers, seeds, buds, leaves, herbs, wood, fruits, twigs, and roots [74]. EOs demonstrate broad-spectrum antimicrobial properties and are generally considered safe for human and animal consumption [74, 75]. Different microbes react differently to EOs by either promoting or inhibiting specific groups of microorganisms such as methanogens [76]. Some inhibit the growth of protozoa indirectly or by biohydrogenation of unsaturated fatty acids limiting the hydrogen availability for methanogens [77, 78]. Below is a tabulated summary of the effects of essential oils from various plants such as garlic, eucalyptus, clove, rosemary, thyme, paprika, juniper, ginger in vitro, and in vivo below (Table 1).

Different research groups have evaluated the efficacy of secondary metabolites, including saponins, flavonoids, tannins, and other terpenoids [70]. Guyader *et al*. [95] observed a reduction in methane emission (29%) and protozoal population by (50%) with an increasing dosage of saponin during an in vitro batch culture. Woodward *et al*. [96] also compared the effect of tannin-containing legume *Lotus pedunculatus* with the ryegrass on sheep moderating methane emission of up to 28% (g/kg DMI). A separate report also demonstrated 50% methane reduction using condensed tannin-containing forage in goats, although it negatively affected other conditions such as total tract protein digestion [97].

### Additional Organic Additives

Biochar has become increasingly popular during the last decade as it has been shown to improve growth, egg yield, blood profiles, inhibitory effects against the growth of rumen pathogens, and reduce enteric methane emission [98, 99]. Seaweeds, also known as macroalgae, including brown (Phaeophyta), red (Rhodophyta), and green (Chlorophyta) seaweeds, have become preferable feed additives because of their anti-methanogenic properties [100, 101]. Several in vitro studies of seaweed supplements showed a negative correlation with methane generation especially using *Asparagopsis taxiformis* [72, 102, 103] and its fellow *Asparagopsis* spp., which could cut back in vivo methane emission from 50% to over 80% in dairy cattle [104-106].

Prebiotics such as chitosan, inulin, and yeast products can also limit enteric methane emissions by modifying the rumen bacterial community structure [107, 108]. Yeast products and inulin stimulate the growth of other rumen bacteria competing for hydrogen against methanogens [109], while chitosan disrupts the cell wall permeability of methanogen causing cell death [110]. However, their usage in ruminants is still relatively limited compared to other feed additives and requires further research to encourage its adoption [111].

### III. Mitigation through Direct-Fed Microbials (DFMs)/Probiotics

DFMs are defined as a single or mixed culture of live organisms, which promotes desirable rumen microflora and provide beneficial effects when fed to animals [112]. Various rumen bacteria are thought to compete with methanogens for the hydrogen supply by promoting propionogenesis, acetogenesis, and nitrate/nitrite or sulfate reduction which can serve as an alternative H_2_ sink. This redirects the metabolic flow of rumen hydrogen towards VFAs production which could otherwise be used for methanogenesis [113].

### Propionic Acid Bacteria (PAB)

Propionibacteria are gram-positive bacteria that naturally inhabit the rumen at approximately 4.3% of the total rumen microbial population. They produce propionate via two pathways: The succinate and acrylate pathway [114-116]. The propionate production process utilizes H_2_ when reducing pyruvate to propionate. Since H_2_ is a limiting substrate for methane production, the inclusion of propionate-forming bacteria as DFMs could lower methane production [117].

Numerous strains of PAB that could play a critical role in reducing methane emissions have been tested in vitro and in vivo. These include *Propionibacterium acidipropionici*, *P. freudenreichii*, *P. propionicus*, *P. jensenii*, *P. japonicas*, and *P. japonicas* [118-120]. Recently, another PAB strain, *Propionibacterium thoenii* T159 has demonstrated 20%methane reduction and a 21% increase in the total VFA production when rumen fluid from Norwegian dairy cows fed with grass silage–concentrate mixture was used in vitro [121]. However in vivo, *Propionibacteria* spp. fails to persist in the rumen of cattle fed with a diet rich in starch. Elevated starch fermentation results in an increased molar proportion of propionate thereby reducing the efficacy of inoculated *Propionibacterium* spp. [118, 120, 122, 123].

### Acetogens

Homoacetogens are a diverse group of 23 different bacterial genera capable of producing acetate [124]. These acetogenic bacteria are present in rumen between 10^7^ to 10^8^ cells/g and grow heterotrophically by utilizing sugars. It can also thrive autotrophically by utilizing H_2_ and CO_2_ [117, 125] catalyzed by a hydrogenase enzyme via the Wood–Ljungdahl (WLP) pathway [126, 127].

Several attempts have already been made to isolate homoacetogens from the rumen and analyze their role as an alternative hydrogen sink, including *Acetitomaculum ruminis*, *Eubacterium limosum*, *Blautia schinkii*, and *Blautia producta* [128]. Furthermore, in vitro studies have also suggested that acetogenesis could serve as an alternative to methanogenesis in eliminating H_2_ from the rumen [129]. However, their abundance and affinity towards hydrogen are generally lower than hydrogenotrophic methanogen [127, 130]. As Lopez *et al*. [130] have concluded, high concentrations of acetogenic bacteria cannot compete against methanogens for H_2_ disposal, making it unclear whether homoacetogens could play a pivotal role in the ruminal ecosystem [128].

### Methane Oxidizing Bacteria (MOB)

MOB is a class of bacteria that can grow on methane as a sole carbon and energy source. It is ubiquitous in either micro-oxic or aerobic environments [131, 132]. These bacteria utilize a specialized enzyme called methane monooxygenase (MMO), which oxidizes methane to methanol [133]. Methanol is then further oxidized to formaldehyde catalyzed by methanol dehydrogenase, then assimilated into the serine or ribulose-5-monophosphate pathway (RuMP) for biomass synthesis [134, 135].

Even though there is a growing number of methane oxidation and MOB enrichment studies from ruminants, the possibility of MOB as a potential probiotic for cattle has hardly received attention from the international scientific community [136].

In 2003, Kajikawa *et al*. [137] used carbon isotope labeling and estimated the flux of ^13^C to CO_2_. Around 0.2-0.5% of methane oxidation was attributable to microbial cells when ^13^CH_4_ was incubated together with mixed rumen microbes from sheep. MOB was also detected in both rumen fluid and rumen epithelium from non-lactating Holstein cows [138]. Moreover, Valdez *et al*. [139] decreased in vitro methane accumulation when MOB isolated from young pigs was used. MOBs have also been successfully enriched and taxonomically characterized as *Methylocystis* and *Methylobacter* from *Bos indicus* steers [136]. Furthermore, Stocks and McCleskey [140] isolated a MOB morphologically and physiologically related to *Methanomonas methanooxidans*. Recently, a group from India also isolated a Ca. *Methylobacter coli* BlB1 from the feces of an Indian antelope that can utilize both methane and methanol [141].

However, in vivo studies using MOBs as probiotics remain scarce. Isolation, screening, and in vivo studies of MOB need to expand to realize its probiotic potential in alleviating methane emissions while enhancing animal nutrition.

## Conclusion

Cattle farming is the single most significant contributor to global methane emissions. As the demand for quality meat and milk products rises, methane emissions and global temperature increase. Therefore, one of the most effective strategies to ameliorate climate change is to subdue ruminant methane emissions. Feed manipulation remains the most cost-effective approach, attaining a substantial 60% reduction in methane just by meticulously selecting the type or quality of forage and optimizing the concentrate to forage ratio in feed. Many organic and inorganic feed additives also hold tremendous potential to attenuate CH_4_ production by directly or indirectly transforming the rumen microbial community. Chemical additives including 3-NOP, ionophores, and halogenated compounds have exhibited exceptional declines in vitro and in vivo rumen methanogenesis; by stimulating the growth of microbes competing for the same substrate used by methanogens or as a direct inhibitor of methanogens. Lately, emphasis on biological feed additives such as essential oils, macroalgae, biochar, and other plant metabolites has grown over human health concerns. Along with is the growing significance of probiotics as feed supplements. In this review, we discussed two DFMs strategies. The one is by using microbes that compete against methanogens for hydrogen availability, such as propionic acid bacteria, acetogens, and nitrate/sulfate-reducing bacteria. Another is using MOB that directly utilize the methane generated during the ruminal fermentation process. The usage of probiotics to tackle climate change carries considerable breadth and depth, but their inability to compete with rumen methanogens for H_2_, or colonize and proliferate in the rumen needs to be addressed. Identifying potential probiotics that can minimize rumen methane generation while maintaining a balanced gastrointestinal ecosystem remains the most attractive strategy. To conclude, developing an efficient and effective methane mitigation strategy while improving animal performance is critical in achieving agricultural sustainability.

## Figures and Tables

**Table 1:** Effect of essential oils from various plant sources on methane emission.

Plant source	Effect on methane emissions	Reference
Garlic	91% reduction in CH_4_ production (in vitro)	[79]
	73% reduction in CH_4_ production (in vitro)	[80]
	Improved feed digestibility in dairy cows	[81]
Thyme	30% reduction in CH_4_ production (in vitro)	[82]
	21% reduction in CH_4_ production in cows	[83]
	Increased propionate production in Holstein calves	[84]
Rosemary	Over 20% reduction in CH_4_ production (in vitro)	[85]
	9% reduction in CH_4_ production (in vitro)	[86]
Oregano	87% reduction in CH_4_ production (in vitro)	[87]
	11% reduction in CH_4_ production (in vitro)	[88]
Clove	34% reduction in CH_4_ production (in vitro)	[89]
	No effect on CH_4_ production in dairy cows	[90]
Eucalyptus	Up to 85% reduction in CH_4_ production (in vitro)	[91]
	No effect on CH_4_ production in sheep	[92]
Lavender	Up to 60% reduction in CH_4_ production (in vitro)	[93]
	54% reduction in CH_4_ production (in vitro)	[94]
Peppermint	Over 30% reduction in CH_4_ production (in vitro)	[95]
	52% reduction in CH_4_ production (in vitro)	[96]
